# Keratinocyte growth factor impairs human thymic recovery from lymphopenia

**DOI:** 10.1172/jci.insight.125377

**Published:** 2019-06-20

**Authors:** Alasdair J. Coles, Laura Azzopardi, Onajite Kousin-Ezewu, Harpreet Kaur Mullay, Sara A.J. Thompson, Lorna Jarvis, Jessica Davies, Sarah Howlett, Daniel Rainbow, Judith Babar, Timothy J. Sadler, J. William L. Brown, Edward Needham, Karen May, Zoya G. Georgieva, Adam E. Handel, Stefano Maio, Mary Deadman, Ioanna Rota, Georg Holländer, Sarah Dawson, David Jayne, Ruth Seggewiss-Bernhardt, Daniel C. Douek, John D. Isaacs, Joanne L. Jones

**Affiliations:** 1 Department of Clinical Neurosciences, University of Cambridge, Cambridge, United Kingdom.; 2 Nuffield Department of Clinical Neurosciences and; 3 Department of Paediatrics, University of Oxford, Oxford, United Kingdom.; 4 Cambridge Clinical Trials Unit, Cambridge University Hospitals NHS Foundation Trust, Cambridge, United Kingdom.; 5 Medical Research Council (MRC) Biostatistics Unit, Cambridge Institute of Public Health, Cambridge, United Kingdom.; 6 University Hospital of Würzburg, Würzburg, Germany.; 7 Department of Hematology/Oncology, Soziastiftung Bamberg, Bamberg, Germany.; 8 National Institute of Allergy and Infectious Diseases, NIH, Bethesda, Maryland, USA.; 9 Institute of Cellular Medicine, Newcastle University, and Musculoskeletal Unit, Newcastle upon Tyne Hospitals NHS Foundation Trust, Newcastle upon Tyne, United Kingdom.

**Keywords:** Immunology, Therapeutics, Autoimmune diseases, T cell development, T cells

## Abstract

**BACKGROUND:**

The lymphocyte-depleting antibody alemtuzumab is a highly effective treatment for relapsing-remitting multiple sclerosis (RRMS); however, 50% of patients develop novel autoimmunity after treatment. Most at risk are individuals who reconstitute their T cell pool by proliferating residual cells, rather than producing new T cells in the thymus, raising the possibility that autoimmunity might be prevented by increasing thymopoiesis. Keratinocyte growth factor (palifermin) promotes thymopoiesis in nonhuman primates.

**METHODS:**

Following a dose tolerability substudy, individuals with RRMS (duration ≤10 years; expanded disability status scale ≤5.0, with ≥2 relapses in the previous 2 years) were randomized to placebo or 180 μg/kg/d palifermin, given for 3 days immediately before and after each cycle of alemtuzumab, with repeat doses at month 1 (M1) and M3. The interim primary endpoint was naive CD4^+^ T cell count at M6. Exploratory endpoints included number of recent thymic emigrants (RTEs) and signal joint T cell receptor excision circles/ml (sjTRECs/ml) of blood. The trial’s primary endpoint was incidence of autoimmunity at M30.

**RESULTS:**

At M6, individuals receiving palifermin had fewer naive CD4^+^ T cells (2.229 × 10^7^/l vs. 7.733 × 10^7^/l; *P* = 0.007), RTEs (16% vs. 34%), and sjTRECs/ml (1100 vs. 3396), leading to protocol-defined termination of recruitment. No difference was observed in the rate of autoimmunity between the 2 groups.

**CONCLUSION:**

In contrast with animal studies, palifermin reduced thymopoiesis in our patients. These results offer a note of caution to those using palifermin to promote thymopoiesis in other settings, particularly in the oncology/hematology setting, where alemtuzumab is often used as part of the conditioning regime.

**TRIAL REGISTRATION:**

ClinicalTrials.gov NCT01712945.

**FUNDING:**

MRC and Moulton Charitable Trust.

## Abstract 2

Keratinocyte growth factor impairs T cell recovery in patients with relapsing-remitting multiple sclerosis receiving treatment with the lymphocyte-depleting monoclonal antibody alemtuzumab.

## Introduction

T cell lymphopenia is strongly associated with autoimmunity (1–5). A striking example is autoimmunity following treatment of relapsing-remitting multiple sclerosis (RRMS) with the lymphocyte-depleting, humanized anti-CD52 mAb alemtuzumab (Lemtrada). Two short courses of alemtuzumab given 12 months apart effectively suppress RRMS for many years (6–10); however, between 6 months and 5 years after treatment, 40% of patients develop thyroid autoimmunity (typically Graves’ disease). A further 2% of individuals develop idiopathic thrombocytopenic purpura, and 0.1% anti–glomerular basement membrane disease, and rare cases of autoimmune hemolytic anemia, autoimmune neutropenia, and autoimmune pancytopenia have been reported. An additional 20% of patients develop novel autoantibodies without clinical symptoms (6–8, 11–13).

We have previously shown that, although B cell reconstitution after alemtuzumab is rapid, via the generation of new cells from the bone marrow (14), CD4^+^ and CD8^+^ T cells take 35 and 20 months, respectively, to reach normal range (15). Furthermore, and paradoxically, for at least 9 months after treatment, thymopoiesis (determined by measuring naive T cell production, recent thymic emigrants, and T cell receptor excision circles) is reduced (16). Instead, T cell reconstitution occurs by the proliferation of cells that have escaped depletion. As a result, the after treatment T cell pool is dominated by “memory-like cells” with a restricted T cell receptor (TCR) repertoire (16). In keeping with animal studies demonstrating the proautoimmune nature of lymphopenia-induced T cell proliferation (2–4, 17), we have shown that individuals with the least thymic function and most restricted TCR repertoire after alemtuzumab are at the greatest risk of developing autoimmune complications (16). These observations raised the possibility that autoimmunity after alemtuzumab might be reduced if thymic function could be restored.

Keratinocyte growth factor (KGF) promotes thymopoiesis through its trophic effects on thymic epithelial cells (TECs). TECs play a pivotal role in T cell development, providing essential growth factors and presenting self-antigen to developing thymocytes. When administered to mice undergoing bone marrow transplantation or experimental graft-versus-host disease (GvHD), KGF enhanced thymopoiesis (18, 19). In rhesus macaques, KGF enhanced thymic naive T cell production and reduced lymphopenia-induced T cell proliferation after myeloablation and peripheral blood progenitor cell autologous transplantation (20). In this model, KGF (given as palifermin at a dose of 250 μg/kg per day for 3 days before and after transplantation) was well tolerated, and its positive effects on the thymus were maintained for up to 12 months. In 2005, palifermin was licensed (as Kepivance) to prevent mucositis induced by chemotherapy. In its pivotal trial, 60 μg/kg palifermin was given for 3 days before conditioning and then for 3 days after hematopoietic stem cell transplantation (HSCT; ref. 21); this regimen was well tolerated. Later, a trial of 3 doses of palifermin (60 μg/kg) before conditioning and up to 9 doses after allogeneic HSCT showed the treatment to be safe, although it had no effect on the incidence of acute GvHD (22) or absolute lymphocyte count recovery (23). Although thymic function was not directly studied in these patients, the result suggested that higher doses of palifermin might be required to see positive immunological effects.

Therefore, we designed a study to explore the tolerability of higher doses of palifermin (90, 120, and 180 μg/kg/d, given for 3 days before and after alemtuzumab with further doses at months 1 and 3) and then test the efficacy of the highest tolerated dose in a placebo-controlled trial (CAMTHY) aimed at testing 2 hypotheses: (a) palifermin increases thymic T cell reconstitution after alemtuzumab and (b) thereby reduces the risk of alemtuzumab-induced autoimmunity. Here we report the unexpected results of a preplanned interim analysis (aimed at testing hypothesis 1) that led to protocol-defined termination of recruitment.

## Results

### Clinical trial participants.

**Figure 1:**
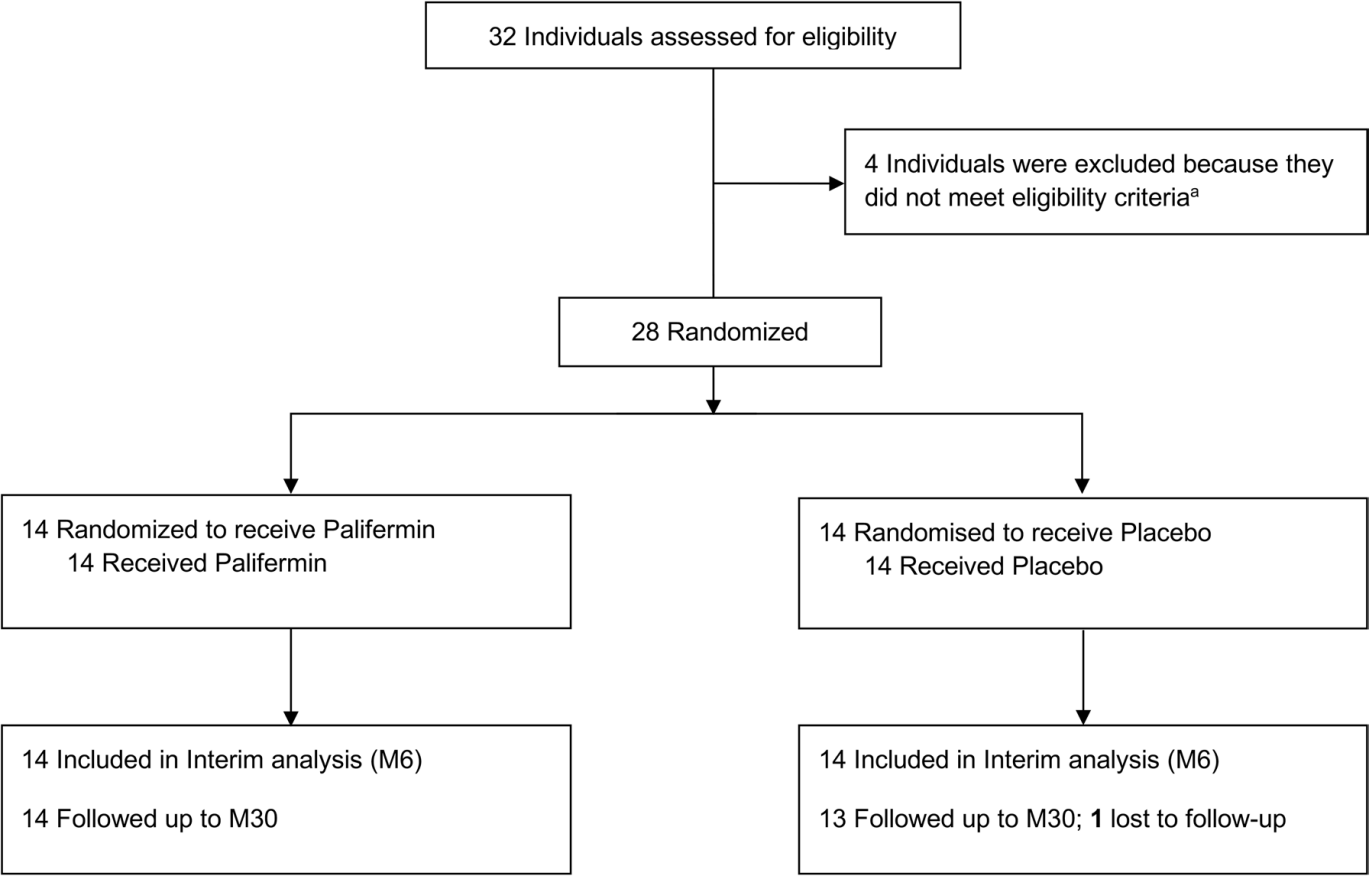
Flow of participants randomized to palifermin (rhKGF) versus placebo on CAMTHY. One individual was excluded because their RRMS disease activity was insufficient to warrant treatment with alemtuzumab; 2 individuals were excluded because of abnormal liver function tests; 1 individual was excluded because of abnormal thyroid function tests.

Between June 2013 and February 2015, 28 patients were enrolled and randomized to 1 of 2 treatment groups (palifermin, *n* = 14; placebo, *n* = 14), then followed for 30 months (Figure 1). Their baseline characteristics are shown in Table 1. The preplanned interim analysis was conducted by independent statisticians and the unblinded results were reported first to the trial steering committee, who made the decision to recommend early termination of the trial, as per protocol; no further patients were recruited and no further palifermin was given. All enrolled patients completed the study, and all analyses were completed with blinding intact, after which 1 investigator (AJC) was unblinded.

### Palifermin significantly reduced thymic T cell reconstitution in our patient cohort.

**Figure 2:**
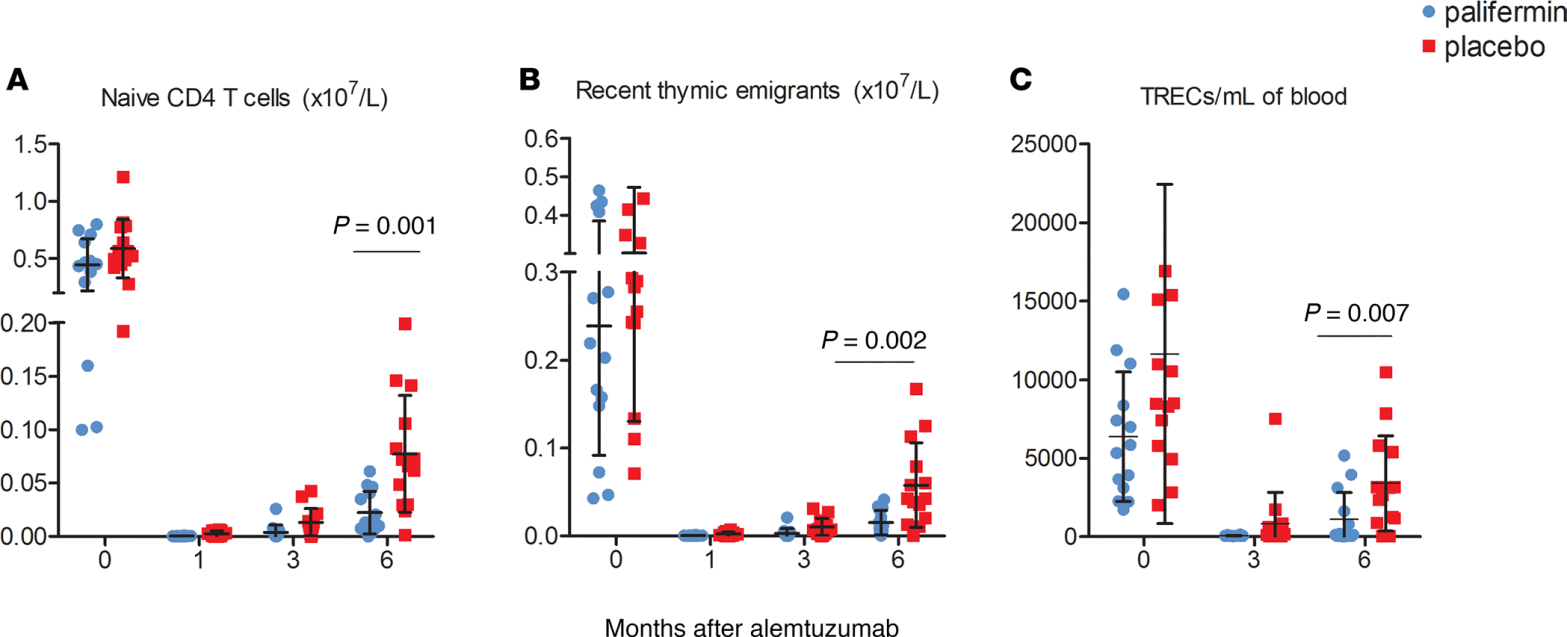
Thymic function is significantly reduced in patients treated with palifermin (*n* = 14) versus placebo (*n* = 14). (**A**) Numbers of circulating naive (CCR7^+^CD45RA^+^) CD4^+^ T cells. (**B**) Numbers of circulating CD4^+^ recent thymic emigrants (RTEs), defined as naive CD4^+^ T cells coexpressing CD31. (**C**) T cell receptor excision circles (TRECs) per ml of blood, at baseline (0) and months 3 and 6 after alemtuzumab. The data shown are the mean ± SD; *P* values are shown for the month 6 data and were calculated using Mann-Whitney nonparametric tests. Naive CD4^+^ cell count at month 6 was the predefined interim primary outcome measure. *P* values for RTEs and TRECs are shown for descriptive purposes only.

As we have previously reported (16), thymic function (assessed by measuring naive T cells, recent thymic emigrants [RTEs], and T cell receptor excision circles/ml [TRECs/ml]) was significantly reduced following treatment with alemtuzumab. However, unexpectedly, this was further impaired in patients receiving palifermin. The interim analysis endpoint, mean naive (CCR7^+^CD45RA^+^) CD4^+^ T cell count at month 6, was reduced in the palifermin group: 2.23 × 10^7^/l (SD 2.0) versus 7.73 × 10^7^/l (SD 5.74) in those receiving placebo (*P* = 0.007; Figure 2A and Supplemental Table 1; supplemental material available online with this article; https://doi.org/10.1172/jci.insight.125377DS1), even after adjusting for baseline naive CD4^+^ T cell counts, total palifermin dose, and age (adjusted treatment group *P* = 0.007; Supplemental Table 2). This difference was also evident at months 1 and 3 after treatment: 0.036 × 10^7^/l (SD 0.025) versus 0.341 × 10^7^/l (SD 0.25) and 0.387 × 10^7^/l (SD 0.68) versus 1.326 × 10^7^/l (SD 1.29), respectively (Supplemental Table 1). The difference in naive CD4^+^ T cell numbers was greatest at month 1, suggesting that palifermin’s negative effect on thymic function occurred early. This was not due to globally reduced T cell numbers but due to a specific reduction in naive T cells (Supplemental Table 1).

Palifermin also reduced the mean proportion of RTEs in the CD4^+^ T cell pool — month 1, 2.94% (SD 2.77) versus 7.93% (SD 8.71); month 3, 4.83% (SD 7.88) versus 13.29% (SD 11.75); and month 6, 16.05% (SD 13.21) versus 33.95% (SD 18.68; Figure 2B and Supplemental Table 1). TRECs/ml were also lower in the palifermin group at months 3 and 6, with median values 54.64 and 130.42 versus 162.93 and 2900.99, respectively (Figure 2C and Supplemental Table 3). In keeping with reduced thymopoiesis, there was a trend toward more restricted CD4^+^ and CD8^+^ TCR repertoires after palifermin; for instance, Shannon’s entropy was 12.7 versus 13.3 in the placebo group, and the mean CD4 clonality score was 0.102 versus 0.067. Palifermin reduced the number of unique clones/μg of DNA (75,111 versus 84,017; Supplemental Tables 4 and 5). As per our previous reports, the CD8^+^ TCR repertoire was more restricted than the CD4^+^ TCR repertoire at baseline, becoming increasingly restricted after treatment, particularly in the palifermin-treated group (Supplemental Tables 4 and 5).

Following alemtuzumab, mean proportions of T effector memory RA cells and in particular effector memory cells were increased in the CD4^+^ T cell pool, particularly in the palifermin arm (Supplemental Table 1). Similar changes were seen in the CD8^+^ T cell pool (Supplemental Table 6). Palifermin had no effect on the usual rise in the relative number of CD4^+^ T regulatory cells (CD4^+^CD25^hi^CD127^lo^) after alemtuzumab (Supplemental Table 7). No difference was seen in thymic size or density between the 2 arms of the study (Supplemental Table 8).

In view of the unexpected negative effects of palifermin on thymopoiesis, we retrospectively assessed thymic function in patients treated during the dose escalation substudy. Naive CD4^+^ T cells and TRECs/ml at 6 months were lower in the 90 μg/kg arm of the dose escalation (*n* = 3) compared with placebo and lower still in the 3 patients on 120 μg/kg or 180 μg/kg palifermin (Supplemental Tables 9 and 10). Given the variation in TRECs/ml between individuals before treatment, we normalized TRECs/ml at 6 months to baseline levels (Supplemental Table 10); TRECs/ml was lowest — at 9.64% of baseline — in the 180 μg/kg group versus 13.57% after 120 μg/kg and 28.89% following 90 μg/kg palifermin.

### Adverse events.

Adverse events were common in both arms of the study (Table 2). In keeping with the chemotherapy experience, palifermin caused an infusion syndrome comprising an erythematous rash, edema of the hands and face, oral symptoms (sensory and/or altered taste), and discoloration of the tongue. Unexpectedly, 10 of 14 patients treated with palifermin developed transient hair thinning (lasting weeks to months) after treatment; in 1 individual, this was marked. Mild to moderate upper respiratory tract infections were also more common in the palifermin group, occurring in 8 of 14 patients versus 4 of 14 patients in the control arm. Palifermin administration before alemtuzumab did not alter the latter’s well-reported infusion-associated symptoms, except that chest tightness was reported less commonly. At the interim analysis cutoff (month 6 for each participant), no serious adverse events/suspected unexpected serious adverse reactions were reported.

### Autoimmunity was not increased by palifermin.

Although the protocol-defined early termination of the trial meant it was underpowered to detect an effect of palifermin on the development of autoimmunity, patients were categorized at month 30 into those who had developed a clinical autoimmune disease during the trial, those who had developed novel autoantibodies (measured on at least 2 occasions 6 months apart) without developing clinical symptoms, and those with no expression of autoimmunity. There were no differences between the groups. Four of 14 patients on palifermin developed a clinical autoimmune disease, compared with 5 of 13 on placebo (1 patient was lost to follow-up in the placebo group; Fisher’s exact test, 2-sided; *P* = 0.69). Five of 14 patients on palifermin developed either clinical autoimmunity or de novo autoantibodies, compared with 8 of 13 on placebo (*P* = 0.26).

### Human and murine TECs express CD52.

**Figure 3:**
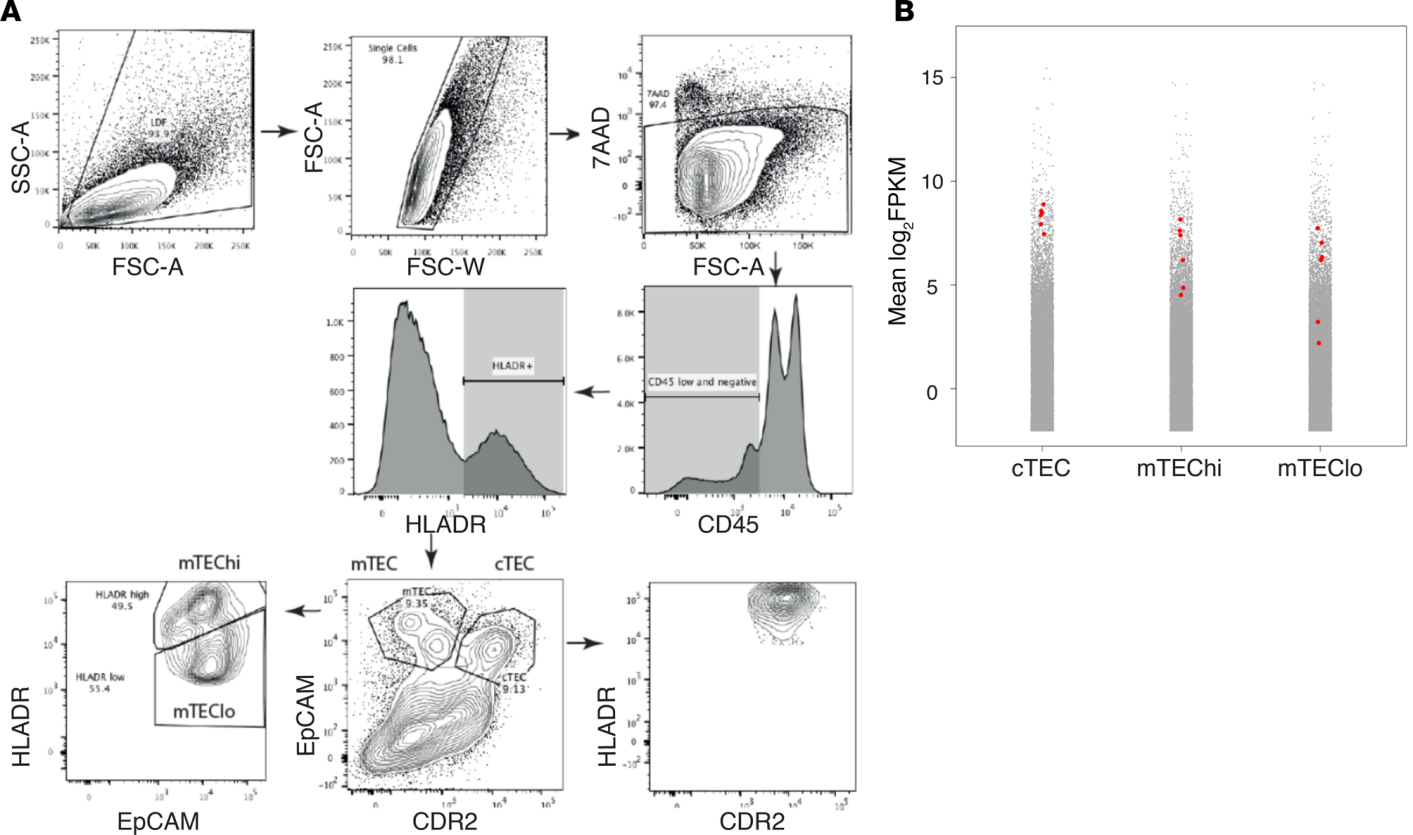
Analysis of *CD52* expression in human thymus. (**A**) FACS strategy for isolating TEC subsets: cTEC as EpCAM^lo^ CDR2^+^, mTEC^lo^ as EpCAM^hi^ CDR2^–^ MHC^lo^, and mTEC^hi^ as EpCAM^hi^ CDR2^–^ MHC^hi^. FSC-A, forward scatter area; FSC-W, forward scatter width; 7-AAD, 7-amino-actinomycin D. (**B**) *CD52* expression in RNA-Seq data sets from human thymus (*n* = 6). Plots show the distribution of gene expression (mean fragments per kilobase of transcript per million mapped reads [FPKM]) in cTEC, mTEC^hi^, and mTEC^lo^. Red dots represent individual sample levels of CD52 expression; gray dots are mean expression, across all samples, of all other genes.

Recently published RNA-Seq data have shown that murine cortical and medullary TECs (cTECs and mTECs) express CD52, alemtuzumab’s target molecule, at least at the level of mRNA (24, 25). Given this, we analyzed bulk RNA-Seq data, generated from sorted human cTECs and mTECs collected in the Holländer Lab in Oxford (see Methods), and found that CD52 is also highly expressed by human TECs (Figure 3; Gene Expression Omnibus [GEO] accession GSE127209). *CD52* expression was within the top 10% of detectable transcripts in all TEC subsets and was significantly higher in cTEC than mTEC^lo^ (3.8-fold change, adjusted *P* = 0.02) but not between cTEC and mTEC^hi^ or mTEC^hi^ and mTEC^lo^ (adjusted *P* > 0.05 in both).

## Discussion

Here we report the unexpected finding that palifermin (KGF) exacerbates alemtuzumab’s negative effect on thymopoiesis. We have demonstrated this by 3 independent techniques: naive CD4^+^ T cell count (the primary interim outcome measure) and circulating numbers of RTEs and TRECs/ml. Because the overall aim of the trial was predicated on palifermin’s ability to boost thymopoiesis to reduce autoimmunity after alemtuzumab, in accordance with the trial protocol, recruitment to the study was halted and further dosing of palifermin suspended following a planned interim analysis.

Our results contradict palifermin’s ability to enhance thymopoiesis in murine and nonhuman primate models (18–20). Although a species difference is possible, the fact that the KGF receptor (FGFR2IIIb) is expressed on human epithelial cells makes this unlikely. We also do not believe that this is a dose effect. Our decision to test the efficacy of the highest tolerated dose of palifermin was based on our interpretation of results from a trial of palifermin in preventing GvHD following allogeneic HSCT. In that study, 3 daily doses of 60 μg/kg palifermin before conditioning and up to 9 doses after transplant did not accelerate total lymphocyte recovery or reduce the incidence of acute GvHD. Although the absence of detailed immune phenotyping data and lack of information on TRECs and TCR repertoire make it difficult to distinguish palifermin’s effect on the thymus versus lymphopenia-induced proliferation, the result suggested to us that 60 μg/kg was unlikely to have a positive effect on thymopoiesis. Our suspicion was confirmed by the results of another trial of palifermin, published during the course of this study, which demonstrated that up to 3 doses of 60 μg/kg palifermin increased neither CD4^+^ T cell counts nor thymic function (assessed by measuring naive CD4^+^ cells, RTEs, and thymic size on CT scan) in HIV-infected patients with persistent CD4^+^ T cell lymphopenia despite virologically effective antiretroviral treatment (26); suboptimal dosing was postulated as a cause for the negative result. Importantly, no previous study to our knowledge has reported a reduction in thymic function with palifermin. In our own study, none of the doses tested in the tolerability substudy had a positive effect on thymopoiesis. With the caveat that only 3 individuals were treated at each dose level, all doses (from 90 to 180 μg/kg/d) impaired thymic function after alemtuzumab, with an apparent dose effect.

By analyzing publically available (24, 25) and in-house–generated RNA-Seq data sets (GEO accession GSE127209), we have shown that murine and human TECs express high levels of *CD52* mRNA, with *CD52* appearing in the top 10% of all detectable transcripts in all TEC subsets. Interestingly, in the human thymus, cTECs express the highest level of *CD52* of the TEC subsets, whereas in mouse thymus, cTECs express the lowest level of *Cd52* (24). Species differences are therefore clearly important when considering the potential effects of anti-CD52 treatment on thymopoiesis. High levels of *CD52* mRNA in TECs raise the possibility that alemtuzumab impairs thymic function by damaging CD52-expressing TECs. Studies using the human CD52-transgenic mouse model (27) confirm that alemtuzumab can penetrate the thymus, causing depletion of CD52-expressing single-positive and double-positive thymocytes (TECs were not analyzed in this study). However, thymic T cell depletion was significantly less than in the periphery (50% of thymocytes at a dose of 10 mg/kg vs. 100% T cell depletion in the circulation at 0.5–1 mg/kg), suggesting that antibody penetrance is incomplete.

Given this, we hypothesize that palifermin may worsen alemtuzumab’s negative effect on thymic function by causing TECs to upregulate CD52 expression, making them more susceptible to damage. Although density of CD52 expression is not the only factor that determines susceptibility to alemtuzumab-induced depletion, it is a critical factor (28, 29). In support of this, in our study, palifermin’s negative effect on thymic function was most marked at the earliest time points, at the point of coadministration with alemtuzumab. For example, the biggest difference in the number of naive CD4^+^ T cells between the 2 arms of the study was at month 1, where there was a 9.5-fold difference compared with a 3.4-fold difference at month 3 and a 2.6-fold difference at month 6. A similar effect was seen in the TREC/ml data, where the biggest difference between the 2 arms of the study was at month 3 (the earliest point measured; a 13-fold difference versus a 3-fold difference at month 6). These data suggest that although the initial doses of palifermin exaggerate alemtuzumab-induced thymic damage, later doses may be protective.

We have previously reported that reduced thymic function and consequent lymphopenia-induced T cell proliferation is greatest in those who develop autoimmunity after alemtuzumab (16). Our new finding of TEC *CD52* expression also raises the possibility that alemtuzumab leads to autoimmune complications because of a direct effect on central tolerance, as has been reported in a murine model of acute GvHD, where GvHD-induced mTEC^hi^ loss resulted in reduced tissue-restricted self-antigen expression and de novo generation of autoreactive T cells (30).

Although palifermin significantly reduced thymopoiesis in our patients, there was no evidence that it increased the risk of developing autoimmunity at 30 months of follow-up. However, autoimmunity can occur for up to 5 years after alemtuzumab, so we will continue to monitor these patients clinically and immunologically. Eight of 14 patients treated with palifermin developed mild to moderate upper respiratory tract infection compared w/ 4 of 14 patients in the control arm, perhaps reflecting an increased susceptibility to infective complications. However, urinary tract infections were equal in both groups (2 of 14), and no serious infections occurred in either arm of the study. Furthermore, infections were not more common in those receiving the higher dose of palifermin in the open-label dose escalation study compared with the other doses — 2/3 versus 0/3 versus 1/3 in the low-, middle-, and high-dose groups, respectively (Supplemental Table 11).

In conclusion, we have shown that palifermin (180 μg/kg/d given over 12 days) worsens thymic function following alemtuzumab treatment of RRMS and therefore should not be used to promote T cell reconstitution in this setting. Although we hypothesize that palifermin’s negative impact on thymopoiesis is due to coadministration with alemtuzumab, our trial serves as a note of caution to those performing or contemplating trials of palifermin in other settings, particularly in the oncology/hematology setting, where alemtuzumab is often used as part of the conditioning regime. We also note that palifermin worsened clinical outcome in the KARE study (31), a trial of KGF in the treatment of acute respiratory distress syndrome, despite encouraging results in animal studies. Together, KARE and CAMTHY serve as a reminder to be cautious when translating efficacy data from animal studies to humans and when coadministering drugs that may interact. It remains to be seen whether alemtuzumab-induced autoimmunity can be reduced by preserving thymic function.

## Methods

### Participants.

Participants were aged 18 to 50 years with RRMS (32), disease duration of 10 years or less, at least 2 relapses in the previous 2 years with at least 1 in the previous 12 months (untreated or on β-interferon or glatiramer acetate), and an expanded disability status scale score of 5.0 or less. Exclusion criteria included progressive forms of multiple sclerosis; previous thymectomy; previous treatment with alemtuzumab, natalizumab, mitoxantrone, cyclophosphamide, cladribine, rituximab, or any other immunosuppressant or cytotoxic therapy; and a history of malignancy or a history of clinically significant autoimmunity other than multiple sclerosis.

### Randomization and masking.

Participants were randomized (1:1) to receive palifermin or placebo using an online randomization service. Because palifermin’s known adverse effects (skin reddening and tongue discoloration) may compromise blinding, samples for immunological assays were recoded with a randomly generated identifier for each participant visit and were analyzed while blinded in batches. Radiological assessments of thymic size and density were performed by masked assessors outside the core trial team.

### Drug treatments.

All patients received 12 mg/d alemtuzumab for 5 consecutive days at baseline, followed by 12 mg/d for 3 consecutive days at month 12, with methylprednisolone pretreatment on days 1, 2, and 3 of each cycle. As is standard practice, all patients were given 200 mg oral acyclovir twice a day for 28 days after each cycle of alemtuzumab to reduce the risk of oral herpes simplex.

For the open-label dose escalation tolerability substudy, 3 individuals were treated at each of the following palifermin doses: 90 μg/kg/d, 120 μg/kg/d, and 180 μg/kg/d given as an intravenous bolus injection on days –5, –4, and –3 before each cycle of alemtuzumab and on days 8, 9, and 10. Three further doses were given at month 1 (±7 days) and month 3 (±2 weeks) after each cycle of alemtuzumab. Each dose level was separated by a minimum of 10 days (from the day 10 dose), and escalation between doses occurred only if no adverse events greater than grade 2 occurred. Because all doses were equally tolerated (Supplemental Table 11), for the subsequent placebo-controlled study, participants received 180 μg/kg/d of palifermin, or an equivalent volume of normal saline.

### Clinical and laboratory assessments.

In addition to standard alemtuzumab safety monitoring, at each 3-month visit for 30 months of follow-up, participants were assessed clinically and their blood assayed for markers of thymic function by immune phenotyping, for signal joint TRECs (sjTRECs) in whole blood quantification (33), and for TCRβ chain sequencing. In addition, to assess thymic size and density, a noncontrast, low-dose chest CT scan was performed at baseline and at month 6. Details of these methods are as follows.

### Immunophenotyping.

Peripheral blood mononuclear cells were isolated from heparinized blood by density centrifugation on Ficoll. For surface staining, washed cells were resuspended in FACS buffer (PBS, 2 mM EDTA, 0.01% sodium azide) containing 2% mouse serum for 20 minutes to reduce nonspecific binding, incubated at 4 degrees for 30 minutes with different mAb combinations against surface targets, washed twice in FACS buffer, and then fixed for 20 minutes in 2% formaldehyde before acquisition. The FoxP3/Transcription Factor Staining Buffer set (eBioscience) was used for intracellular staining of FoxP3 and Ki67. Cells were fixed and made permeable for 40 minutes at room temperature and then stained for 30 minutes at room temperature in permeabilization buffer (Invitrogen). The following fluorescent-labeled antibodies (all purchased from BD Biosciences) were used in various combinations (catalog numbers are given in parentheses): CD3-V450 (560365), CD3-APCCy7 (557832), CD4-V500 (560768), CD8-APC (555369), CCR7-FITC (561271), CD45RA-PECy5 (555490), CD45RA-PECy7 (337186), CD25-PE (555432), Ki67-PE (556027), CD127-V450 (560823), CD31-V450 (561653), and FoxP3-AF647 (560045). Data were acquired on a Canto II (BD Biosciences) and analyzed using FlowJo v7.6.5 (Tree Star Inc.); the gating strategy is outlined in Supplemental Figure 1. Cell counts for the different CD4 and CD8 subpopulations were calculated based on CD4 and CD8 counts determined by FACS performed at the Department of Immunology, Addenbrooke’s Hospital, Cambridge, United Kingdom — a clinical laboratory approved for good clinical practice. Where CD4 or CD8 counts were below the laboratory detectable limit, the value was replaced in the analysis with the lower limit of detection divided by the square root of 2 (LLD/√2).

### sjTREC/ml quantification.

Thymic function was also estimated by quantification of sjTRECs in whole blood as described by Lorenzi et al. (33). In brief, DNA was extracted directly from 300 μl of fresh blood collected in EDTA using the Wizard Genomic DNA purification kit (Promega). The TREC content of each sample was determined by quantitative PCR using a standard curve derived from plasmid constructs encoding the sjTREC sequence. Standards were diluted over the range 10^7^ to 10^1^, and a curve was run in each experiment alongside positive and negative controls. TaqMan technology was used in a 25-μl reaction mixture containing 700 nM of each primer CACATCCCTTTCAACCATGCT and GCCAGCTGCAGGGTTTAGG, 150 nM TaqMan hydrolysis probe (6-FAM-ACACCTCTGGTTTTTGTAAAGGTGCCCACT-TAMRA), and 12.5 μl JumpStart Taq ReadyMix (MilliporeSigma), supplemented with MgCl_2_ at a final concentration of 5 mM. Each reaction contained 500 ng DNA. Samples were run in triplicate and the replicate average taken as the sample result. Cycling conditions were 94°C 2 minutes, 40 cycles at 94°C 30 s/60°C 15 s/72°C 2 min, and 72°C 5 minutes. The number of sjTRECs/ml was calculated as: total DNA (μg) in 300 μl whole blood/DNA (μg) in PCR reaction (0.5 μg) × no. of TRECs (derived from the standard curve) × 1000/300. Primers and probes were all from MilliporeSigma.

### TCR repertoire analysis.

Genomic DNA from magnetically sorted CD4 and CD8 cells was extracted using the Qiagen Allprep method, according to the manufacturer’s instructions. Samples were quantified and diluted for library preparation. Amplification and sequencing of CDR3 regions in rearranged TCRβ chains were performed using the immunoSEQ assay (Adaptive Biotechnologies). The immunoSEQ assay combines multiplex PCR with high-throughput sequencing and a sophisticated bioinformatics pipeline for TCRβ CDR3 region analysis. Sequencing data were analyzed using the immunoSEQ Analyzer (https://clients.adaptivebiotech.com/login). Sample diversity or “richness” was calculated by Shannon’s entropy, defined as:

 (Equation 1),
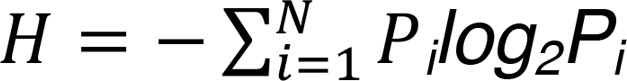


where *n* represents the number of unique clones and *P*_i_ represents the frequency of the clones *i*. Entropy ranges from 0, in a sample with only 1 clone, to *H_max_* = log_2_*N*, for polyclonal, highly diverse samples. Clonality was defined as 1-Pielou’s evenness metric and was calculated using 1 – *H*/ln(*N*). Clonality describes the shape of the distribution of proportional abundances and ranges from 0 to 1; values near 1 indicate an increasingly asymmetric division in which a few clones are present at high frequencies (34, 35).

### Thymic imaging.

To assess thymic size and density, a noncontrast, low-dose chest CT scan was performed at baseline and at 6 months on a Siemens Emotion 16 (2007) scanner, with contiguous 1-mm sections. Measurements of thymus height, width, depth (cm), and volume (cm^3^) of the thymus and average density (measured in Hounsfield units) were made on the soft tissue window reconstructions using Siemens Syngo via multimodality reading solution imaging software. A region-of-interest volume tool was used that involved manual contouring of the thymus on several transaxial levels, with semi-automated propagation (Supplemental Figure 2). Transaxial measurements were made using a caliper measuring tool (Siemens syngo.via software). All measurements were performed by 1 of 2 radiologists, 1 a thoracic radiology consultant (10 years’ experience) and the other a specialty registrar who had received training. Ten studies were dual read by both radiologists together to ensure consistency in method of measurement, and there was good interobserver agreement. Both radiologists were blinded to the treatment status of the patients, and the same radiologist interpreted both the baseline and repeat CT on each patient.

### Analysis of CD52 expression by human TECs.

Thymus tissue was removed from patients (*n* = 4) aged between 11 days and 3 months old and prepared as described with some modifications (36). In brief, tissue was dissected and physically dissociated in sterile PBS. A single-cell suspension was prepared using 3 rounds of mechanical and enzymatic digestion with Liberase TM/DNAse Ι as detailed in Stoeckle et al. (36). The subsequent antigen-presenting cell–enriched single-cell suspension was enriched for CD4^lo/–^ cells using magnetic-activated cell sorting. cTECs were isolated as EpCAM^lo^ CDR2^+^ and mTECs as EpCAM^hi^ CDR2^–^. mTECs were subsequently separated into MHC^hi^ (mTEC^hi^) and MHC^lo^ (mTEC^lo^). For 3 patients, 30,000–50,000 cells were sorted from each TEC subset. For 1 patient, 3 replicates of 7575 cells were sorted from each TEC subset. The study of human thymus tissue has been granted ethical approval and is publicly listed (Integrated Research Application System [IRAS] ID 156910, Central Portfolio Management System [CPMS] ID 19587).

RNA was extracted from sorted TEC subsets using a Qiagen Plus RNeasy Micro Kit. Smarter-Seq was used to generate transcriptomic libraries, which were subsequently sequenced on an Illumina HiSeq 4000 (37). Adapters were trimmed from reads using Trimmomatic (38). Reads were aligned against the Ensembl human reference genome (GRCh38) using 2-pass mapping with STAR (version 2.5.3a) (39). Aligned reads were assigned to genes using HTSeq (version 0.5.4) (40). EdgeR trimmed mean of M-values was used to adjust counts data before log_2_ FPKM calculation and differential expression analysis (41). *P* values were adjusted for multiple hypothesis testing using Benjamini-Hochberg correction. The vioplot package in R was used to generate plots.

### Statistics.

A “stop-go” interim analysis, testing the effect of palifermin on naive T cell reconstitution (as a readout of thymic function), was planned when 28 patients reached month 6. An independent trial steering committee adjudicated the results of the interim analysis.

The preplanned efficacy threshold for the interim analysis was a statistically significant increase in the number of peripheral naive (CCR7^+^CD45RA^+^) CD4^+^ T cells in the palifermin group, by at least 50%, compared with placebo at month 6 after alemtuzumab (as an indicator of thymopoiesis). We believed this to be a conservative estimate because palifermin increases naive CD4^+^ T cell numbers 3-fold in rhesus macaques and 2-fold in mice (maximal at 3–9 months in macaques and 30–80 days in mice). Power calculations suggested that 28 patients (14 placebo, 14 palifermin) had 80% power to detect this increase. A *P* value of less than 0.05 was considered significant.

Multivariate linear regression was used to model naive CD4^+^ T cell count at 6 months with explanatory variables of treatment group, age, baseline naive CD4^+^ T count, and total dose of palifermin received. To aid interpretation of the model intercept, the continuous variables were median centered. An unpaired, 2-tailed *t* test and Mann-Whitney *U* test were also performed on naive CD4^+^ T cell count at 6 months, comparing palifermin versus placebo. For exploratory endpoints, summary statistics were calculated by treatment arm; no formal statistical tests were applied (exploratory endpoint *P* values reported in the text are given for descriptive purposes only). Continuous variables were summarized using *n* (nonmissing sample size), mean, SD, median, maximum, and minimum. Categorical variables were reported as frequency and percentages (based on the nonmissing sample size) of observed levels. For any laboratory tests where the measurement made was considered less than the detectable limit, the value was replaced in the analysis with the LLD divided by the square root of 2 (LLD/√2).

If the interim analysis had been successful, 80 patients would have been recruited to the trial, which would have given 78% power to detect a relative risk reduction of 50% of autoimmunity after alemtuzumab, using a 2-sided 5% significance level.

### Study approval.

CAMTHY was conducted in accordance with the International Conference on Harmonisation Guidelines for Good Clinical Practice and the principles of the Declaration of Helsinki and was approved by National Research Ethics Service (NRES) Committee London – Hampstead (12/LO/0393). All participants gave written informed consent. The study of human thymus tissue has been granted ethical approval (IRAS ID 156910, CPMS ID 19587). Consent was taken from parents or guardians preoperatively.

## Author contributions

AJC and JLJ conceived the trial. JLJ was chief investigator. AJC, LA, OKE, JWLB, and EN were subinvestigators and alongside JLJ were responsible for the day-to-day running of the trial. HKM, SAJT, LJ, JD, SH, ZGG, SM, IR, MD, AEH, and GH performed and analyzed the laboratory assays. JB and TJS performed and analyzed the thoracic CT scans. SD was the trial statistician. DCD and RSB gave advice on trial design. With the help of DCD, RSB, and DJ, JDI led the adjudication of the interim analysis. AJC, LA, OKE, HKM, SAJT, LJ, JD, SH, DR, JB, TJS, JWLB, EN, KM, ZGG, AEH, SM, MD, IR, GH, SD, DJ, RSB, DCD, JDI, and JLJ revised the manuscript and read and approved the final version.

## Supplementary Material

Supplemental data

ICMJE disclosure forms

## Figures and Tables

**Table 2:**
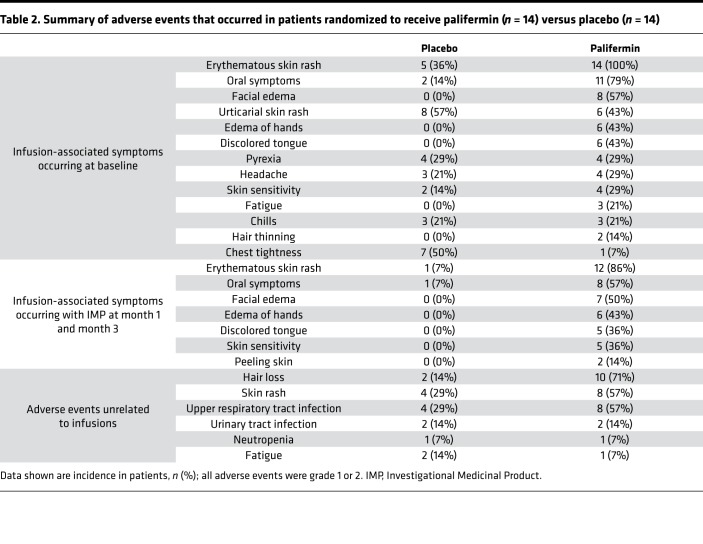
Summary of adverse events that occurred in patients randomized to receive palifermin (*n* = 14) versus placebo (*n* = 14)

**Table 1:**
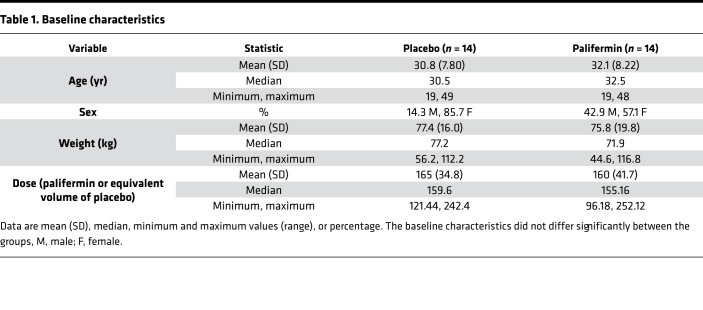
Baseline characteristics

## Version 1 05/07/2019

In-Press Preview

## Version 2 06/20/2019

Electronic publication
