# Supplementary material for: Keratinocyte growth factor impairs human thymic recovery from lymphopenia
Source: JCI Insight. 2019 Jun 20;4(12):e125377. doi: 10.1172/jci.insight.125377 (PMC6629095; doi:10.1172/jci.insight.125377)
Supplement: Supplemental data [file jciinsight-4-125377-s184.pdf]

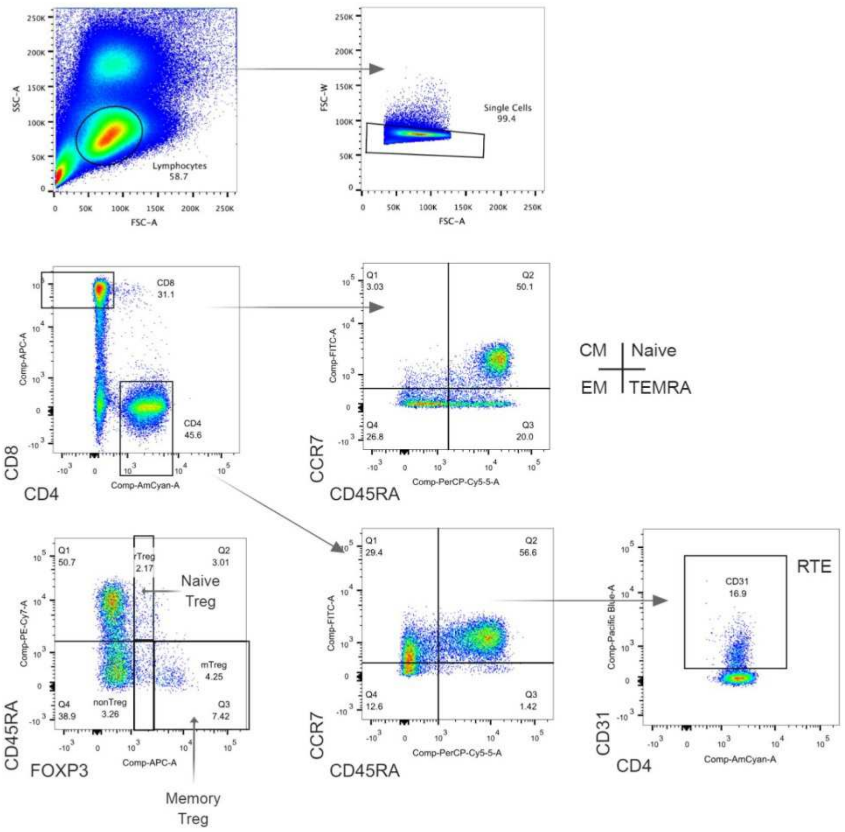

Supplemental Figure 1: Flow cytometry gating strategy. After gating on single lymphocytes CD4 and CD8 T-cells were divided into naïve, central memory (CM), effector memory (EM) and TEMRA populations based on CCR7 and CD45RA expression (naïve CCR7+CD45RA+; CM CCR7+CD45RA-, EM CCR7-CD45RA- and TEMRA CCR7-CD45RA+). The CD4 naïve (CCR7+CD45RA+) population was further split into recent thymic emigrants (RTEs) and non-RTEs based on expression of CD31 (RTE CD4+CCR7+CD45RA+CD31+). CD4 T regulatory cells (Tregs) were divided into naïve and memory subpopulations based on CD45RA and FOXP3 expression (naïve Tregs CD4+CD45RA+FOXP3+; memory Tregs CD4+CD45RA-FOXP3++).

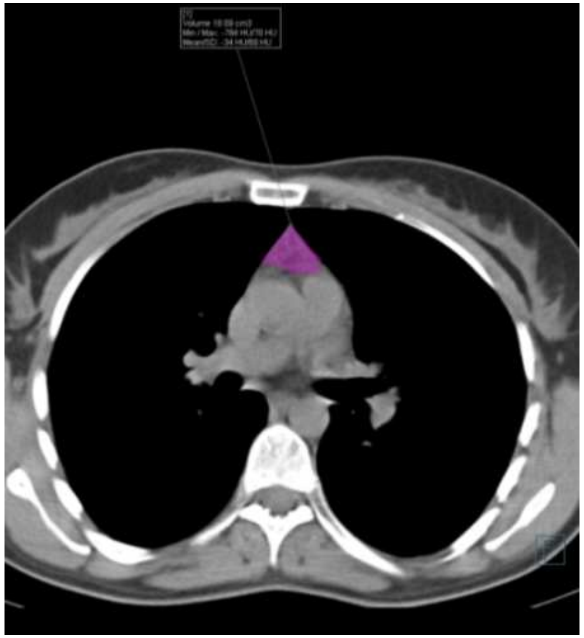

Supplemental Figure 2: Example of CT Chest Imaging – showing the thymus in the anterior mediastinum, with the superimposed region of interest volume tool (purple).

Variable	Group	Statistic	Screening	Baseline	M1	M3	M6
**Sample Numbers**	Placebo	Total Cell count n	14	14	10	14	14
	Palifermin		14	14	8	14	14
	Placebo	% count n	14	13	14	14	14
	Palifermin		14	13	14	14	14
	Placebo	Subset count n	14	13	10	14	14
	Palifermin		14	13	8	14	14
**Total CD4 Count ($x10^9/L$)**	Placebo	Mean (SD)<br>Median<br>Min, Max	0·8907 (0·2774)<br>0·855<br>0·46, 1·32	0·9786 (0·3633)<br>0·855<br>0·68, 1·97	0·0500 (0·0492)<br>0·03<br>0, 0·14	0·0757 (0·0390)<br>0·08<br>0·03, 0·15	0·1493 (0·0664)<br>0·145<br>0·06, 0·31
	Palifermin	Mean (SD)<br>Median<br>Min, Max	0·8043 (0·2981)<br>0·79<br>0·38, 1·38	0·8643 (0·3559)<br>0·895<br>0·35, 1·74	0·0825 (0·1853)<br>0·015<br>0, 0·54	0·0929 (0·0932)<br>0·065<br>0·03, 0·35	0·1064 (0·0551)<br>0·085<br>0·04, 0·25
**% of CD4 that are naïve (CCR7+CD45RA+)**	Placebo	Mean (SD)<br>Median<br>Min, Max	59·61 (14·28)<br>61·55<br>37·4, 85·7	60·30 (14·60)<br>61·6<br>26, 80·9	8·69 (9·89)<br>4·47<br>0·81, 31·6	17·88 (15·93)<br>12·975<br>0·3, 53·5	48·36 (22·24)<br>55·35<br>2·29, 78·6
	Palifermin	Mean (SD)<br>Median<br>Min, Max	47·52 (15·36)<br>50·9<br>23·5, 72·4	48·61 (17·19)<br>45·9<br>23·8, 78·6	3·21 (3·41)<br>2<br>0, 11·2	6·19 (9·78)<br>2·405<br>0·086, 37·1	23·42 (18·77)<br>22·45<br>0·21, 51·8
**Naïve CD4 (CCR7+CD45RA+) count ($x10^7/L$)**	Placebo	Mean (SD)<br>Median<br>Min, Max	53·869 (22·245)<br>52·561<br>18·538, 90·52	59·777 (26·108)<br>56·63<br>19·24, 121·155	0·341 (0·252)<br>0·3092<br>0, 0·67	1·326 (1·293)<br>1·0904<br>0·009, 4·28	7·733 (5·473)<br>6·929<br>0·1603, 19·933
	Palifermin	Mean (SD)<br>Median<br>Min, Max	39·726 (21·395)<br>34·9265<br>10·906, 77·556	44·333 (23·646)<br>45·126<br>9·996, 79·866	0·036 (0·038)<br>0·02485<br>0, 0·1114	0·387 (0·677)<br>0·15195<br>0·0224, 2·597	2·229 (1·997)<br>1·357<br>0·0189, 6·108
**% of CD4 that are RTEs (CD31+CCR7+CD45RA+)**	Placebo	Mean (SD)<br>Median<br>Min, Max	29·86 (13·29)<br>27·9<br>11, 59·6	30·31 (11·57)<br>34·5<br>10·3, 48·4	7·93 (8·71)<br>4·105<br>0·73, 28·9	13·29 (11·75)<br>9·88<br>0·17, 38·9	33·95 (18·68)<br>32·75<br>0·85, 63
	Palifermin	Mean (SD)<br>Median<br>Min, Max	21·32 (9·45)<br>19·85<br>9·03, 40·3	26·44 (12·83)<br>24·8<br>10·2, 48·9	2·94 (2·77)<br>2·26<br>0·034, 11	4·83 (7·88)<br>1·89<br>0·079, 29·8	16·05 (13·21)<br>14·7<br>0·54, 37·1
**RTEs (CD31+CCR7+CD45RA+) count ($x10^7/L$)**	Placebo	Mean (SD)<br>Median<br>Min, Max	13·005 (7·696)<br>12·348<br>2·66, 27·951	15·292 (10·371)<br>12·74<br>3·09, 41·34	3·316 (9·004)<br>0·398<br>0, 28·9	1·336 (2·181)<br>0·8859<br>0, 8·507	5·029 (7·011)<br>3·688<br>0·0085, 28·567
	Palifermin	Mean (SD)<br>Median<br>Min, Max	8·896 (4·239)<br>8·7775<br>2·7993, 16·523	13·229 (8·722)<br>13·184<br>2·144, 33·25	0·115 (0·146)<br>0·05145<br>0, 0·3861	0·238 (0·321)<br>0·08847<br>0·006, 1·023	·1·317 (1·282)<br>09<br>0·1242, 4·83
**% of CD4 that are CM (CCR7+CD45RA-)**	Placebo	Mean (SD)<br>Median<br>Min, Max	26·2 (11·95)<br>25<br>2·38, 46·4	25·1 (11·25)<br>23·9<br>0·81, 46·6	14·9 (12·31)<br>10·8<br>1·11, 48·3	17·3 (7·15)<br>16·7<br>4·27, 33·4	18·5 (8·96)<br>17·35<br>1·64, 36·3
	Palifermin	Mean (SD)	30·2 (7·16)	28·8 (7·27)	21·4 (13·79)	16·6 (10·09)	21·7 (9·21)

		Median	29·1	29·3	18·1	17·65	24·5
		Min, Max	20, 45·5	14·5, 42·7	2·06, 56·6	1·81, 36·1	5·32, 36
**CM CD4 (CCR7+CD45RA-) count (x10^7/L)**	Placebo	Mean (SD)	22·683 (9·464)	24·458 (11·432)	0·616 (0·674)	1·335 (0·799)	2·694 (1·526)
		Median	22·365	24·182	0·377	1·344	2·5045
		Min, Max	1·2614, 40·392	0·567, 42·946	0, 1·932	0·1281, 3·006	0·0984, 6·897
	Palifermin	Mean (SD)	24·617 (11·528)	25·849 (14·258)	0·430 (0·332)	1·123 (0·617)	2·094 (0·959)
		Median	23·61	24·411	0·403	1·398	2·1235
		Min, Max	9·156, 49·595	8·19, 52·548	0, 1·1124	0·0932, 1·88	0·3584, 3·904
**% of CD4 that are EM (CCR7-CD45RA-)**	Placebo	Mean (SD)	12·1 (6·65)	12·1 (7·25)	64·3 (21·63)	56·4 (21·18)	27·5 (21·33)
		Median	11·55	9·66	70·75	60·3	20·8
		Min, Max	1·67, 26·2	0·84, 26·2	4·27, 85·3	12·6, 83·4	5·72, 71·1
	Palifermin	Mean (SD)	19·5 (9·77)	19·2 (11·24)	66·4 (14·47)	67·9 (17·04)	45·8 (19·97)
		Median	16·05	19·1	65·55	74·35	45·45
		Min, Max	6·49, 38·9	5·66, 39	39·8, 96·4	29·7, 84·6	17·1, 75·2
**EM CD4 (CCR7-CD45RA-) count (x10^7/L)**	Placebo	Mean (SD)	10·89 (7·30)	12·33 (9·01)	3·66 (4·40)	4·39 (2·88)	3·89 (3·00)
		Median	9·9209	10·1926	1·452	3·616	3·184
		Min, Max	0·8851, 28·82	0·588, 30·732	0, 11·718	0·378, 9·698	0·3432, 10·849
	Palifermin	Mean (SD)	14·40 (5·85)	15·22 (9·29)	7·60 (17·99)	6·08 (5·86)	4·97 (4·35)
		Median	14·042	13·65	1·0295	4·206	3·998
		Min, Max	3·4272, 24·035	3·8976, 39·324	0, 52·056	2·079, 21·97	1·791, 18·8
**% of CD4 that are TEMRA (CCR7-CD45RA+)**	Placebo	Mean (SD)	2·07 (2·61)	2·55 (4·58)	12·13 (15·41)	8·40 (10·98)	5·64 (10·76)
		Median	1·425	1·28	8·435	4·805	2·295
		Min, Max	0·095, 10·2	0·2, 17·5	2·79, 63	0, 43·6	1·1, 42·7
	Palifermin	Mean (SD)	2·76 (3·30)	3·39 (4·21)	9·00 (8·32)	9·30 (12·85)	9·16 (16·47)
		Median	1·165	1·41	6·38	5·21	3·425
		Min, Max	0·54, 10·2	0·66, 12·4	1·2, 26·7	1·09, 51	0·68, 64·6
**TEMRA CD4 (CCR7+CD45RA+) count (x10^7/L)**	Placebo	Mean (SD)	1·638 (1·641)	2·203 (3·206)	0·388 (0·355)	0·522 (0·425)	0·611 (0·633)
		Median	1·2433	1·5192	0·3325	0·435	0·46875
		Min, Max	0·07505, 5·406	0·1725, 12·25	0, 1·26	0, 1·308	0·077, 2·562
	Palifermin	Mean (SD)	1·704 (1·494)	2·074 (1·922)	0·182 (0·277)	1·699 (4·684)	1·352 (3·087)
		Median	1·06615	1·1875	0·0819	0·29	0·28695
		Min, Max	0·252, 5·304	0·6204, 7·192	0, 0·8316	0·0763, 17·85	0·0544, 11·628

Supplemental Table 1: Characterisation of the CD4 population into naïve (CD45RA+CCR7+), central memory (CD45RA-CCR7+), effector memory (CD45RA-CCR7-) and TEMRA (CD45RA+CCR7-) subsets. Data show either cell subset count or percentage of the total CD4+ T cell population. Data show: mean (SD), median and minimum and maximum values.

Variable	Estimate	95% confidence interval	p-value
Intercept	5·294	0·948, 9·64	0·027
Treatment: Placebo vs. Palifermin	-5·132	-8·3, -1·964	0·005
Age (years)	-0·151	-0·353, 0·051	0·158
Baseline Naïve CD4+ T-cell count	0·051	-0·016, 0·118	0·154
Total Placebo/Palifermin dose	0·011	-0·033, 0·055	0·621

Supplemental Table 2: Results of multivariate linear regression. Where age, baseline naive CD4 count and total Palifermin dose are median-centred, the intercept represents the estimated naive CD4 T-cell count at 6 months for a 31 year old patient receiving Placebo, with a baseline CD4 T-cell count of 4 and a total Palifermin dose of 155mg.

Variable	Group	Statistic	Screening	Baseline	M3	M6
TRECs/mL	Placebo	n<br>Mean (SD)<br>Median<br>Min, Max	14<br>14498·2 (10500·8)<br>12145·767175<br>2182·447437, 44661·9694	14<br>11650·6 (10804·1)<br>8508·5298895<br>2012·348712, 45769·11665	14<br>846·1 (1980·6)<br>162·92944065<br>49·93802302, 7530·082989	14<br>3395·5 (3038·8)<br>2900·9870755<br>56·82513607, 10497·06299
	Palifermin	n<br>Mean (SD)<br>Median<br>Min, Max	14<br>11567·4 (8593·3)<br>9802·973622<br>1720·08455, 30307·69819	13<br>6735·9 (4055·6)<br>5855·968675<br>2235·920272, 15441·01851	14<br>64·6 (27·9)<br>54·636672535<br>42·2178011, 150·4546818	14<br>1100·1 (1721·6)<br>130·4216061<br>25·34423421, 5179·969108

Supplemental Table 3: T cell receptor circles/mL of blood. Data show: n, mean (SD), median, minimum and maximum values (range).

Variable	Group	Statistic	Screening	M6
**Sample Numbers**	Placebo	n	13	14
	Palifermin	n	14	14
**CD4 Clonality**	Placebo	Mean (SD)<br>Median<br>Min, Max	0·0375 (0·00781)<br>0·03585857<br>0·03017301, 0·05763811	0·0669 (0·03475)<br>0·054343265<br>0·03364677, 0·14011244
	Palifermin	Mean (SD)<br>Median<br>Min, Max	0·0638 (0·07221)<br>0·04150275<br>0·03352844, 0·31023574	0·1022 (0·08390)<br>0·079028835<br>0·03899742, 0·3464845
**CD4 Shannon's entropy**	Placebo	Mean (SD)<br>Median<br>Min, Max	15·2 (0·18)<br>15·139041<br>14·77885, 15·378655	13·3 (1·50)<br>13·712763<br>10·31376, 15·0130625
	Palifermin	Mean (SD)<br>Median<br>Min, Max	14·6 (1·47)<br>15·07127225<br>9·58258, 15·196354	12·7 (2·09)<br>13·555009<br>8·372956, 14·92308
**Number of unique clones/ ug DNA**	Placebo	Mean (SD)<br>Median<br>Min, Max	139536 (13669)<br>141958<br>117395, 168923	84017 (36989)<br>93483·5<br>9743, 126215
	Palifermin	Mean (SD)<br>Median<br>Min, Max	123118 (27336)<br>129185<br>38020, 150158	75111 (29936)<br>84755<br>20999, 124700
**Total frequency of top (%) 20 clones**	Placebo	Mean (SD)<br>Median<br>Min, Max	1·37 (1·23)<br>0·90441766<br>0·51445915, 4·775824	5·00 (5·44)<br>2·527990275<br>0·68910601, 19·11048208
	Palifermin	Mean (SD)<br>Median<br>Min, Max	4·86 (9·76)<br>1·64020026<br>0·66854091, 38·0358239	9·78 (10·98)<br>5·602800835<br>1·39888024, 39·84258683

Supplemental Table 4: CD4 clonality data. Data show: n, mean (SD), median, minimum and maximum values (range).

Variable	Group	Statistic	Screening	M6
**Sample Numbers**	Placebo	n	13	14
	Palifermin	n	14	14
**CD8 Clonality**	Placebo	Mean (SD)<br>Median<br>Min, Max	0·136 (0·0721)<br>0·10035078<br>0·04902521, 0·25257745	0·172 (0·1273)<br>0·10737668<br>0·05752725, 0·40934533
	Palifermin	Mean (SD)<br>Median<br>Min, Max	0·138 (0·0768)<br>0·10242532<br>0·06491759, 0·27877614	0·264 (0·1526)<br>0·2728574<br>0·07362837, 0·5184298
**CD8 Shannon's entropy**	Placebo	Mean (SD)<br>Median<br>Min, Max	13·11 (1·29)<br>13·44983<br>11·284007, 14·839041	10·90 (2·95)<br>11·64596375<br>3·4748309, 13·79748
	Palifermin	Mean (SD)<br>Median<br>Min, Max	13·02 (1·40)<br>13·662123<br>10·533361, 14·5808325	9·48 (2·93)<br>9·8403895<br>5·365707, 13·506013
**Number of unique clones/ ug DNA**	Placebo	Mean (SD)<br>Median<br>Min, Max	94054 (25106)<br>94902·5<br>56125, 127720	46209 (26569)<br>39843·02607<br>9580, 95197·5
	Palifermin	Mean (SD)<br>Median<br>Min, Max	89672 (21385)<br>88376·25<br>52742·5, 123575	30890 (25455)<br>24710<br>5650, 76360
**Total frequency of top (%) 20 clones**	Placebo	Mean (SD)<br>Median<br>Min, Max	13·8 (9·15)<br>8·92213444<br>3·37677901, 29·1517279	24·6 (23·32)<br>11·764251335<br>3·81559545, 81·99052133
	Palifermin	Mean (SD)<br>Median<br>Min, Max	14·3 (10·19)<br>9·67576011<br>4·81115464, 32·95189837	35·6 (23·70)<br>31·433465595<br>6·91309173, 75·30927941

Supplemental Table 5: CD8 clonality data. Data show: n, mean (SD), median, minimum and maximum values (range).

Variable	Group	Statistic	Screening	Baseline	M1	M3	M6
**Sample Numbers**	Placebo	Total Cell count n	14	14	10	14	14
	Palifermin		14	14	8	14	14
	Placebo	% count n	14	13	14	14	14
	Palifermin		14	13	14	14	14
	Placebo	Subset count n	14	13	10	14	14
	Palifermin		14	13	8	14	14
**Total CD8 Count ($x10^9/L$)**	Placebo	Mean (SD)<br>Median<br>Min, Max	0·4229 (0·180)<br>0·385<br>0·19, 0·77	0·4979 (0·262)<br>0·42<br>0·22, 1·06	0·2460 (0·337)<br>0·045<br>0, 1	0·0957 (0·128)<br>0·06<br>0, 0·47	0·1321 (0·131)<br>0·095<br>0·01, 0·53
	Palifermin	Mean (SD)<br>Median<br>Min, Max	0·4443 (0·202)<br>0·4<br>0·14, 0·93	0·5279 (0·326)<br>0·475<br>0·16, 1·33	0·1500 (0·295)<br>0·04<br>0, 0·87	0·1864 (0·328)<br>0·07<br>0·01, 1·26	0·1743 (0·189)<br>0·08<br>0·02, 0·68
**Percentage of CD8 that are naïve (CCR7+CD45RA+)**	Placebo	Mean (SD)<br>Median<br>Min, Max	50·72 (14·45)<br>50·3<br>11·8, 68	46·92 (11·78)<br>48·4<br>24·2, 64·1	7·47 (17·42)<br>1·57<br>0, 67·1	17·86 (17·97)<br>12·35<br>0·34, 66·5	37·19 (22·19)<br>36·25<br>6·77, 64·9
	Palifermin	Mean (SD)<br>Median<br>Min, Max	44·69 (17·64)<br>50·35<br>13·7, 65·8	40·67 (18·16)<br>44·2<br>9·87, 65·6	1·87 (2·63)<br>0·835<br>0, 9·83	13·30 (19·02)<br>2·29<br>0·069, 60·8	23·70 (25·43)<br>12·2<br>0·25, 67·8
**Naïve CD8 (CCR7+CD45RA+) count ($x10^7/L$)**	Placebo	Mean (SD)<br>Median<br>Min, Max	19·9996 (7·4873)<br>20·1475<br>8·588, 31·647	22·0470 (10·5024)<br>19·844<br>7·986, 48·23	6·8969 (21·1552)<br>0·0852<br>0, 67·1	1·1369 (1·2838)<br>0·615<br>0, 4·1172	4·1293 (3·4602)<br>4·0995<br>0·0677, 14·204
	Palifermin	Mean (SD)<br>Median<br>Min, Max	19·0920 (10·0442)<br>20·424<br>5·162, 41·344	20·8392 (14·6189)<br>20·992<br>1·5792, 58·786	0·0192 (0·0187)<br>0·01515<br>0, 0·0522	0·5113 (0·5548)<br>0·2631<br>0·00966, 1·824	1·5410 (1·5038)<br>0·74325<br>0·0575, 5·12
**% of CD8 that are CM (CCR7+CD45RA-)**	Placebo	Mean (SD)<br>Median<br>Min, Max	7·42 (6·24)<br>5·99<br>0·087, 20·5	5·37 (5·40)<br>2·75<br>0·17, 17·6	1·74 (1·65)<br>1·535<br>0·19, 6·03	3·15 (3·56)<br>2·235<br>0, 13·4	3·06 (2·75)<br>2·145<br>0·24, 9·28
	Palifermin	Mean (SD)<br>Median<br>Min, Max	7·17 (3·54)<br>6·795<br>3·4, 16	5·15 (3·56)<br>3·08<br>0·93, 13·7	2·07 (2·61)<br>1·005<br>0, 7·14	1·58 (1·73)<br>1·13<br>0·11, 6·32	3·01 (4·40)<br>1·975<br>0·15, 17·6
**CM CD8 (CCR7+CD45RA-) count ($x10^7/L$)**	Placebo	Mean (SD)<br>Median<br>Min, Max	6·1950 (4·8925)<br>5·8579<br>0·0957, 17·63	5·3740 (6·3782)<br>3·08<br>0·119, 24·288	0·0329 (0·0280)<br>0·0277<br>0, 0·0848	0·2006 (0·1692)<br>0·1694<br>0, 0·5832	0·4182 (0·3535)<br>0·2708<br>0·0144, 1·173
	Palifermin	Mean (SD)<br>Median<br>Min, Max	6·0763 (4·4641)<br>4·6126<br>1·292, 17·44	4·9027 (4·6015)<br>2·632<br>0·8835, 15·618	0·0424 (0·0432)<br>0·028<br>0, 0·112	0·0986 (0·0913)<br>0·089<br>0·0063, 0·316	0·2615 (0·3478)<br>0·2198<br>0·0228, 1·408
**% of CD8 that are EM (CCR7-CD45RA-)**	Placebo	Mean (SD)<br>Median<br>Min, Max	21·2 (9·16)<br>21·95<br>1·71, 35·1	26·1 (10·81)<br>23·4<br>0·73, 39·8	59·9 (25·13)<br>70·95<br>1·93, 89·8	45·1 (20·70)<br>48·95<br>2·11, 73·5	27·3 (15·28)<br>25·05<br>2·67, 65·1
	Palifermin	Mean (SD)<br>Median<br>Min, Max	28·3 (8·75)<br>29·3<br>12·6, 43·5	28·2 (10·87)<br>27·7<br>12·9, 44·5	58·9 (22·80)<br>65·6<br>17·2, 86·1	38·9 (16·67)<br>40·65<br>5·31, 70·9	30·9 (17·17)<br>28·75<br>1·65, 77·5

**EM CD8 (CCR7-CD45RA-) count (x10^7/L)**	Placebo	Mean (SD)<br>Median<br>Min, Max	19·08 (8·81)<br>19·267<br>0·9063, 31·35	25·02 (10·76)<br>25·651<br>0·511, 45·126	2·65 (2·65)<br>2·1255<br>0, 7·882	3·26 (1·80)<br>2·587<br>0·0633, 6·708	3·88 (1·80)<br>4·3845<br>0·1602, 5·89
	Palifermin	Mean (SD)<br>Median<br>Min, Max	23·41 (12·42)<br>21·213<br>4·788, 46·644	25·99 (18·73)<br>18·96<br>5·88, 76·734	5·39 (11·81)<br>1·0355<br>0, 34·506	2·58 (1·16)<br>2·149<br>1·38, 5·174	2·92 (1·69)<br>2·551<br>0·297, 6·975
**% of CD8 that are TEMRA (CCR7-CD45RA+)**	Placebo	Mean (SD)<br>Median<br>Min, Max	20·7 (16·9)<br>13·95<br>2·64, 59·7	21·6 (13·1)<br>16·6<br>6·74, 49·9	30·8 (24·8)<br>21·6<br>6·42, 94·3	33·9 (24·0)<br>31·1<br>6·05, 86·7	32·4 (21·8)<br>21·6<br>6·26, 72·2
	Palifermin	Mean (SD)<br>Median<br>Min, Max	19·9 (17·4)<br>14·55<br>4·48, 66·2	26·0 (18·7)<br>20<br>5·3, 69·9	37·2 (23·9)<br>28·7<br>10·9, 82·8	46·2 (25·1)<br>49·1<br>5·28, 94·5	42·4 (27·7)<br>38·6<br>11, 97·6
**TEMRA CD8 (CCR7+CD45RA+) count (x10^7/L)**	Placebo	Mean (SD)<br>Median<br>Min, Max	19·59 (18·21)<br>13·61<br>1·2144, 65·67	23·13 (20·17)<br>12·5718<br>4·9876, 70·723	2·08 (2·70)<br>1·3385<br>0, 7·904	2·86 (3·04)<br>1·6805<br>0·2652, 10·8	4·93 (4·69)<br>3·3155<br>0·626, 16·709
	Palifermin	Mean (SD)<br>Median<br>Min, Max	13·21 (7·55)<br>15·0695<br>3·1746, 25·179	18·89 (9·83)<br>17<br>4·908, 33·858	2·80 (6·71)<br>0·6205<br>0, 19·386	5·87 (9·31)<br>2·553<br>0·264, 33·075	5·25 (6·19)<br>2·44<br>0·88, 20·275

Supplemental Table 6: Characterisation of the CD8 population into naïve (CD45RA+CCR7+), central memory (CD45RA-CCR7+), effector memory (CD45RA-CCR7-) and TEMRA (CD45RA+CCR7-) subsets. Data show either cell subset count or percentage of the total CD4+ T cell population. Data show: mean (SD), median and minimum and maximum values.

Variable	Group	Statistic	Screening	Baseline	M1	M3	M6
**Sample Numbers**	Placebo	Total Cell count n	14	14	10	14	14
	Palifermin		14	14	8	14	14
	Placebo	% count n	14	13	14	14	14
	Palifermin		14	13	14	14	14
	Placebo	Subset count n	14	13	10	14	14
	Palifermin		14	13	8	14	14
**% of CD4 that are Treg**	Placebo	Mean (SD)	5·49 (2·03)	6·58 (1·60)	16·02 (10·70)	15·25 (7·13)	12·31 (6·69)
		Median	6·14	6·86	17·235	15·655	11·155
		Min, Max	0·968, 7·57	3·18, 9·34	0·799, 35·75	0·98, 31·64	3·49, 31·29
	Palifermin	Mean (SD)	5·57 (2·27)	6·48 (1·75)	13·64 (11·96)	16·31 (9·88)	13·61 (5·11)
		Median	5·32	6·29	9·02	14·505	14·845
		Min, Max	1·37, 9·95	4·26, 10·47	1·167, 34·27	2·73, 40·04	4·76, 21·43
**Total Treg count ($x10^7$/L)**	Placebo	Mean (SD)	4·815 (2·290)	6·330 (2·656)	0·401 (0·338)	1·058 (0·643)	1·644 (0·740)
		Median	4·75325	5·321	0·39995	0·87735	1·5665
		Min, Max	0·85184, 8·9326	3·7524, 13·5142	0, 1·0535	0·0784, 2·28	0·6282, 3·2147
	Palifermin	Mean (SD)	4·249 (1·877)	5·443 (2·057)	0·292 (0·242)	1·058 (0·488)	1·285 (0·525)
		Median	4·1357	5·3056	0·30185	1·0815	1·1866
		Min, Max	0·9042, 7·1484	1·7892, 9·6918	0, 0·63018	0·3582, 2·002	0·7524, 2·7344
**naïve Treg count ($x10^7$/L)**	Placebo	Mean (SD)	1·9995 (1·0568)	2·7099 (1·8087)	0·0560 (0·0665)	0·0479 (0·0377)	0·2884 (0·2075)
		Median	1·80205	1·989	0·02213	0·03875	0·23285
		Min, Max	0·6478, 3·9648	0·8296, 7·3481	0, 0·2035	0·007, 0·1128	0·0133, 0·7936
	Palifermin	Mean (SD)	1·7191 (1·1470)	1·7381 (0·8446)	0·0123 (0·0167)	0·0756 (0·0999)	0·1873 (0·2265)
		Median	1·5664	1·653	0·00925	0·0347	0·1215
		Min, Max	0·3154, 4·1322	0·35, 3·393	0, 0·05238	0, 0·35	0·0264, 0·9104
**Memory Treg count ($x10^7$/L)**	Placebo	Mean (SD)	2·815 (1·499)	3·620 (1·396)	0·345 (0·278)	1·010 (0·632)	1·355 (0·666)
		Median	2·84755	3·332	0·3735	0·8564	1·176
		Min, Max	0·08624, 5·31	1·015, 6·1661	0, 0·85	0·036, 2·175	0·3006, 2·4211
	Palifermin	Mean (SD)	2·530 (1·185)	3·705 (1·536)	0·279 (0·231)	0·983 (0·480)	1·098 (0·397)
		Median	2·46805	3·3145	0·2943	1·0115	1·049
		Min, Max	0·2442, 4·4175	1·2054, 6·2988	0, 0·586	0·351, 1·83	0·5994, 1·824

Supplemental Table 7: Characterisation of the CD4+ Treg (CD25hiCD127lo) population. Data show: n, mean (SD), median, minimum and maximum values (range).

Variable	Group	Statistic	Screening	M6
**Thymic volume**	Placebo	n	14	14
		Mean (SD)	8·4 (6·51)	9·1 (7·45)
		Median	8·425	7·305
		Min, Max	0·35, 21·33	0·35, 21·82
	Palifermin	n	14	14
		Mean (SD)	10·2 (7·41)	10·6 (7·73)
		Median	9·325	10·135
		Min, Max	0·35, 24·58	0·35, 23·55
**Thymic density**	Placebo	n	13	13
		Mean (SD)	-2·615 (29·5)	-0·615 (25·3)
		Median	9	-5
		Min, Max	-53, 30	-42, 34
	Palifermin	n	12	12
		Mean (SD)	-22·167 (41·2)	-26·500 (40·4)
		Median	-15	-27·5
		Min, Max	-82, 41	-81, 37

Supplemental Table 8: Thymic size and density. Data show: n, mean (SD), median, minimum and maximum values (range).

Variable	Group	Statistic	Baseline	M1	M3	M6
**Sample Numbers**	Low dose	n	2	3	2	2
	Median dose		3	3	3	3
	High doses		3	2	3	3
**Total CD4 Count (x10^9/L)**	90mcg/Kg	Mean (SD)<br>Median<br>Min, Max	0·870 (0·270)<br>0·740<br>0·69, 1·18	0·065 (0·099)<br>0·010<br>0·006, 0·18	0·130 (0·130)<br>0·060<br>0·05, 0·28	0·127 (0·050)<br>0·120<br>0·08, 0·18
	120mcg/Kg	Mean (SD)<br>Median<br>Min, Max	1·043 (0·463)<br>1·140<br>0·54, 1·45	0·006 (0·00)<br>0·006<br>0·006, 0·006	0·077 (0·046)<br>0·050<br>0·05, 0·13	0·087 (0·064)<br>0·060<br>0·04, 0·16
	180 mcg/Kg	Mean (SD)<br>Median<br>Min, Max	0·88 (0·352)<br>0·920<br>0·51, 1·210	0·024 (0·031)<br>0·006<br>0·006, 0·060	0·039 (0·029)<br>0·050<br>0·006, 0·060	0·057 (0·015)<br>0·060<br>0·04, 0·070
**% of CD4 that are naïve (CCR7+CD45RA+)**	90mcg/Kg	Mean (SD)<br>Median<br>Min, Max	44·65 (0·495)<br>44·650<br>44·3, 45·00	3·55 (2·686)<br>5·020<br>0·45, 5·180	1·62 (0·877)<br>1·620<br>1·00, 2·240	23·45 (11·950)<br>23·450<br>15·0, 31·90
	120mcg/Kg	Mean (SD)<br>Median<br>Min, Max	37·8 (16·441)<br>45·700<br>18·9, 48·80	2·617 (2·724)<br>1·820<br>0·38, 5·65	4·19 (2·745)<br>2·820<br>2·4, 2·35	7·587 (8·325)<br>2·810<br>2·75, 17·20
	180mcg/Kg	Mean (SD)<br>Median<br>Min, Max	30·567 (6·527)<br>27·000<br>26·6, 38·10	1·575 (0·134)<br>1·575<br>1·48, 1·670	7·83 (3·189)<br>8·230<br>4·46, 10·80	10·963 (9·595)<br>7·540<br>3·55, 21·80
**Naïve CD4 (CCR7+CD45RA+) count (x10^7/L)**	90mcg/Kg	Mean (SD)<br>Median<br>Min, Max	42·787 (13·42)<br>42·787<br>33·3, 52·274	0·05492 (0·02467)<br>0·0518<br>0·031947, 0·081	0·2072 (0·1296)<br>0·2072<br>0·1344, 0·28	2·176 (0·5317)<br>2·176<br>1·8, 2·552
	120mcg/Kg	Mean (SD)<br>Median<br>Min, Max	44·3547 (31·0107)<br>52·098<br>10·206, 70·76	0·01665 (0·01733)<br>0·011582<br>0·00242, 0·03596	0·2847 (0·14264)<br>0·3666<br>0·12, 0·3675	0·52813 (0·4661)<br>0·44<br>0·1124, 1·032
	180mcg/Kg	Mean (SD)<br>Median<br>Min, Max	28·114 (16·470)<br>24·472<br>13·77, 46·101	0·04971 (0·05528<br>0·04971<br>0·01063, 0·0888	0·26184 (0·2152)<br>0·223<br>0·0687, 0·4938	0·5376 (0·3296)<br>0·5278<br>0·213, 0·872

Supplemental Table 9: Dose tolerability sub-study CD4 results - characterisation into naïve (CD45RA+CCR7+), central memory (CD45RA-CCR7+), effector memory (CD45RA-CCR7-) and TEMRA (CD45RA+CCR7-) subsets. Data show either cell subset count or percentage of the total CD4+ T cell population. Data show: mean (SD), median and minimum and maximum values.

Variable	Group	Statistic	Baseline	M6
**Sample Numbers**	Low dose	n	3	3
	Median dose		3	3
	High doses		3	3
**TRECS/mL**	90mcg/Kg	Mean (SD)<br>Median<br>Min, Max	8855·98 (5048·31)<br>8601·87<br>3939·52, 14026·54	1590·20 (1104·48)<br>1428·48<br>575·50, 2766·63
	120mcg/Kg	Mean (SD)<br>Median<br>Min, Max	4027·24 (2920·84)<br>5226·54<br>697·65, 6157·54	295·11(258·64)<br>193·75<br>102·51, 589·08
	180 mcg/Kg	Mean (SD)<br>Median<br>Min, Max	3050·29 (1162·30)<br>2863·32<br>1992·81, 4294·75	274·86 (225·87)<br>159·63<br>129·85, 535·10
**TRECS/mL as % of baseline**	90mcg/Kg	Mean (SD)<br>Median<br>Min, Max	-	20·89 (14·82)<br>19·72<br>6·69, 36·26
	120mcg/Kg	Mean (SD)<br>Median<br>Min, Max	-	13·57 (13·20)<br>11·27<br>1·66, 27·77
	180 mcg/Kg	Mean (SD)<br>Median<br>Min, Max	-	9·64 (7·96)<br>6·52<br>3·72, 18·69

Supplemental Table 10: Dose-tolerability sub-study T-cell receptor circles (TRECs)/mL of blood results. Data show: n, mean (SD), median, minimum and maximum values (range).

		90cg/kg/day	120mcg/kg/day	180mcg/kg/day
**Infusion-associated symptoms occurring at baseline**	Erythematous skin rash	3	3	3
	Oral symptoms	3	3	3
	Oedema (facial/hands)	1	3	3
	Urticarial skin rash	1	0	2
	Discoloured tongue	3	3	3
	Pyrexia	3	2	1
	Headache	2	0	2
	Skin sensitivity	0	0	2
	Fatigue	0	0	1
	Chest tightness	2	0	1
	GI upset	1	1	2
**Infusion-associated symptoms occurring with IMP at month 1 and month 3**	Erythematous skin rash	2	3	2
	Oral symptoms	3	3	3
	Oedema (facial/hands)	3	3	3
	Discoloured tongue	3	3	3
	Skin sensitivity	0	0	1
	Peeling skin	0	1	0
	Pyrexia	1	1	0
	GI upset	1	0	1
**AEs unrelated to infusions**	Hair loss	1	1	2
	Dry skin	2	1	2
	Nail changes (ridging)	1	0	1
	Upper respiratory tract infection	2	0	1
	Fatigue	1	0	0
	Low mood	1	1	0

Supplemental Table 11: Adverse event data for sub-study patients only. The data is broken down by the daily dose of Palifermin given (adverse event data for those in the main study, all of whom were treated with 180mcg/kg/day, are shown in table 2). Data are incidence (n). All events were categorised as either severity grade 1 or 2.
